# Cumulative and undiagnosed SARS-CoV-2 infection among the staff of a medical research centre in Tokyo after the emergence of variants

**DOI:** 10.1017/S0950268823000353

**Published:** 2023-03-08

**Authors:** Tetsuya Mizoue, Shohei Yamamoto, Yusuke Oshiro, Natsumi Inamura, Takashi Nemoto, Kumi Horii, Kaori Okudera, Maki Konishi, Mitsuru Ozeki, Haruhito Sugiyama, Nobuyoshi Aoyanagi, Wataru Sugiura, Norio Ohmagari

**Affiliations:** 1 Department of Epidemiology and Prevention, Center for Clinical Sciences, National Center for Global Health and Medicine, Tokyo, Japan; 2 Department of Laboratory Testing, Center Hospital of the National Center for the Global Health and Medicine, Tokyo, Japan; 3 Infection Control Office, Center Hospital of the National Center for the Global Health and Medicine, Tokyo, Japan; 4 Infection Control Office, Kohnodai Hospital of the National Center for the Global Health and Medicine, Chiba, Japan; 5 Center Hospital of the National Center for the Global Health and Medicine, Tokyo, Japan; 6 Kohnodai Hospital of the National Center for the Global Health and Medicine, Chiba, Japan; 7 Center for Clinical Sciences, National Center for Global Health and Medicine, Tokyo, Japan; 8 Disease Control and Prevention Center, National Center for Global Health and Medicine, Tokyo, Japan

**Keywords:** COVID-19, healthcare workers, Japan, SARS-CoV-2, seroprevalence

## Abstract

To describe the trend of cumulative incidence of coronavirus disease 19 (COVID-19) and undiagnosed cases over the pandemic through the emergence of severe acute respiratory syndrome coronavirus 2 (SARS-CoV-2) variants among healthcare workers in Tokyo, we analysed data of repeated serological surveys and in-house COVID-19 registry among the staff of National Center for Global Health and Medicine. Participants were asked to donate venous blood and complete a survey questionnaire about COVID-19 diagnosis and vaccine. Positive serology was defined as being positive on Roche or Abbott assay against SARS-CoV-2 nucleocapsid protein, and cumulative infection was defined as either being seropositive or having a history of COVID-19. Cumulative infection has increased from 2.0% in June 2021 (pre-Delta) to 5.3% in December 2021 (post-Delta). After the emergence of the Omicron, it has increased substantially during 2022 (16.9% in June and 39.0% in December). As of December 2022, 30% of those who were infected in the past were not aware of their infection. Results indicate that SARS-CoV-2 infection has rapidly expanded during the Omicron-variant epidemic among healthcare workers in Tokyo and that a sizable number of infections were undiagnosed.

## Introduction

The pandemic of the coronavirus disease 19 (COVID-19), which is caused by severe acute respiratory syndrome coronavirus 2 (SARS-CoV-2), has continued nearly 3 years despite extensive vaccination rollout. COVID-19 is characterised by a wide variation in clinical manifestation ranging from asymptomatic stage to severe outcome [1], and a sizable portion of the infection are asymptomatic [2]. In Japan, which recorded a relatively low number of patients with COVID-19 in the early period of the pandemic, the epidemic wave has become larger over time, especially after the emergence of the Omicron variants [3]. The reported number of patients diagnosed with COVID-19 are influenced by numerous factors including the availability of and accessibility to the diagnostic test and the presentation of symptoms, which might have changed depending on the epidemic phase and virus type. In this regard, serosurvey is useful to identify past infection including undiagnosed cases [4].

Healthcare workers are thought to be at high risk of SARS-CoV-2 infection. In fact, studies conducted during the early period of the pandemic reported high incidence of COVID-19 [5] and seropositive rate [6] among healthcare workers. Some studies in well-prepared hospitals, however, did not show any difference or even lower seropositive rates relative to the general population among this occupational group. For instance, we previously reported a very low prevalence of SARS-CoV-2 anti-nucleocapsid protein antibody positive before vaccine rollout among the staff of a medical research centre in Tokyo (<1.0% as of December 2020) [7]. Longitudinal data are scarce, however, to show chronologically the expansion of this infection among healthcare workers over the pandemic including post-vaccination, Omicron variant-predominant period. Additionally, while seroepidemiologic studies suggested that a sizable proportion of the infection were undiagnosed [8], it remains elusive whether the proportion of undiagnosed case has changed during the pandemic.

To address these issues, the present extended study was sought to estimate the cumulative SARS-CoV-2 infection and the proportion of undiagnosed infections among healthcare workers based on repeat serological surveys and in-house COVID-19 registry over the Delta- and Omicron-predominant periods.

## Methods

We set-up a repeat serological study in July 2020 among the staff of National Center for Global Health and Medicine (NCGM) [7], which has accepted more than 2000 inpatients with COVID-19 and performed basic and clinical research on COVID-19 since the beginning of the epidemic. As of December 2022, we have completed seven surveys in Toyama (located in central Tokyo, approximately 2500 staff) and three in Kohnodai areas (located in western Chiba, approximately 700 staff). Depending on the target of each survey, the number of participants varied between 943 and 2770, while the participation rate has been high (nearly 80% or above) for all surveys in each area. Participants were asked to donate venous blood and complete a survey questionnaire about COVID-19 diagnosis and vaccine. Written informed consent was obtained from all participants, and the study procedure was approved by the NCGM Ethics Committee (approval number: NCGM-G-003598).

In-house vaccination programme for the NCGM staff started in March 2021. We qualitatively measured immunoglobulin G (IgG) (Abbott ARCHITECT^®^) and total antibodies (Roche Elecsys^®^) against the SARS-CoV-2 nucleocapsid protein at an in-house laboratory according to the manufacturer's instructions. We qualitatively measured antibodies against SARS-CoV-2 nucleocapsid protein using the SARS-CoV-2 IgG assay (Abbott) and Elecsys Anti-SARS-CoV-2 RUO (Roche). The sensitivity and specificity were 100% and 99.9%, respectively, for the Abbott assay [9], and 99.5% and 99.8%, respectively, for the Roche assay [10].

Positive serology was defined as being positive on either or both of these assays. A history of COVID-19 was self-reported and confirmed against the in-house registry. Past infection was defined as either being seropositive at any of the surveys attended or having a history of COVID-19. We calculated the proportion of those infected (cumulative infection) and its 95% confidence interval for all study population as well as their subgroups stratified by sex, age, occupation and SARS-CoV-2 infection risk at work using the exact binomial technique. We also calculated the proportion of undiagnosed infections among those who tested positive. We repeated these analyses only among participants in the Toyama area to confirm the consistency in trend. To examine the discordant serology over time (i.e. positive in Abbott and negative in Roche, and vice versa), we calculated the proportion of such cases.

## Results

Major occupations of the study participants of the survey in June 2022 were nurses (38%), doctors (16%), allied healthcare professionals (13%) and administrative staff (11%). Forty per cent of them reported having engaged in COVID-19-related work after January 2022. Over 90% of the participants in the third survey (June 2021) had completed the second dose of vaccine before the survey (Table 1). The third dose vaccine started in December 2021, and the proportion of the third dose recipients increased from 34% at the fourth survey (December 2021) to 95% at the fifth survey (March 2022). The fourth dose vaccine for healthcare workers in Japan has started in May 2022. The proportion of the fourth dose recipients reached 67% at the last survey (December 2022).

**Table 1.** Chronological change of COVID-19 indicators among the staff of NCGM

	Seroepidemiological surveys						
	1st (July 2020)	2nd (October/December 2020)	3rd (June 2021)	4th (December 2021)	5th (March 2022)	6th (June 2022)	7th (December 2022)
Hospital surveyed	Toyama	Both	Both	Toyama	Toyama	Both	Toyama
Participants, *N*	1228	2564	2770	943	1517	2653	1639
Diagnosed cases^a^, *n* (%)	1 (0.1)	3 (0.1)	18 (0.6)	20 (2.1)	73 (4.8)	252 (9.5)	449 (27.4)
Seropositive cases^b^, *n* (%)	2 (0.2)	18 (0.7)	54 (1.9)	48 (5.1)	121 (8.0)	436 (16.4)	604 (36.9)
Total cases, *n* (%)	3 (0.2)	19 (0.7)	55 (2.0)	50 (5.3)	132 (8.7)	448 (16.9)	639 (39.0)
Awareness^c^, %	33.3	15.8	32.7	40.0	55.3	56.3	70.3
Unawareness^d^, %	66.7	84.2	67.3	60.0	44.7	43.8	29.7
Ratio of total to diagnosed cases	3.0	6.3	3.1	2.5	1.8	1.8	1.4
Vaccination status, %							
Unvaccinated	100	100	7.7	2.8	1.6	1.8	1.2
1- or 2-doses	0	0	92.3	63.2	2.9	4.7	2.8
3-doses	0	0	0	34.0	95.4	92.1	29.4
≥4-doses	0	0	0	0	0.1	1.3	66.5

a Confirmed with PCR or antigen test and/or diagnosed by a physician without test.

b Positive on Abbott and/or Roche SARS-CoV-2 anti-nucleocapsid protein antibody assays.

c Proportion of diagnosed cases among total cases.

d Proportion of undiagnosed cases (seropositive only) among total cases.

**Fig. 1.**
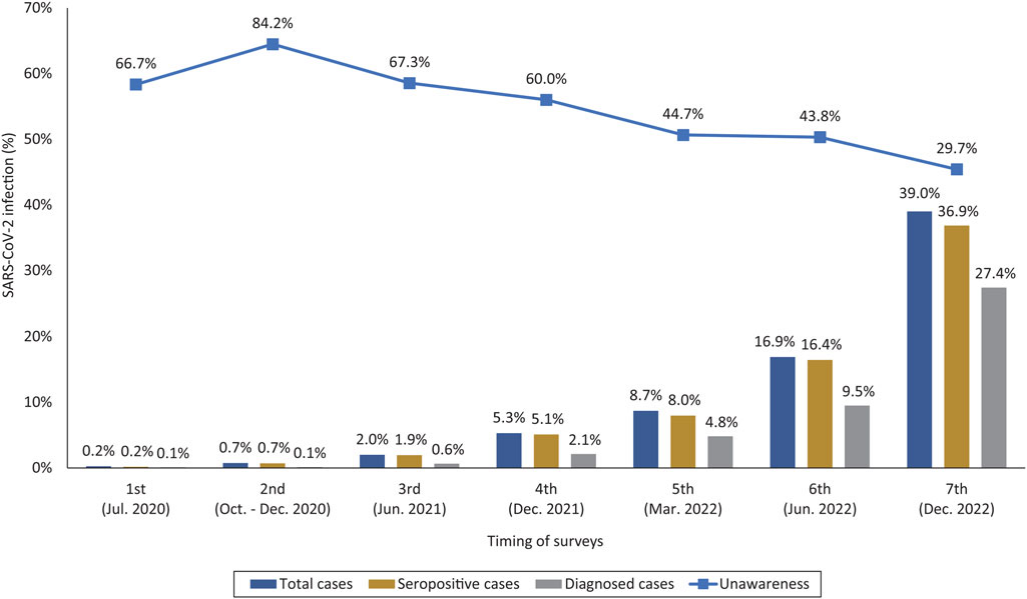
Expansion of SARS-CoV-2 infection among the staff of the NCGM during the pandemic.

Cumulative infection has increased from 2.0% at the third survey (June 2021) to 5.3% at the fourth survey (December 2021), between which the Delta-predominant epidemic occurred (Table 1, Fig. 1). The Omicron (BA.1) predominant epidemic started in January 2022, and the cumulative infection increased to 8.7% as of March 2022. Thereafter, the Omicron BA.1 has been rapidly replaced with the Omicron BA.2 [11]. The cumulative infection reached 16.9% in June 2022. During the summer of that year, Japan was hit by a large epidemic wave, induced predominantly by the Omicron BA.5. As of December 2022, the estimated cumulative infection was 39.0%. The proportion of those who were unaware of their infection has decreased over time. At the latest survey in December 2022, this proportion was 29.7% and the ratio of the number of total infections to the number of known infections (previously diagnosed) was 1.4. The results were similar in the analyses among participants in Toyama area only (Supplementary Table S1).

Before the emergence of the Omicron variants, the SARS-CoV-2 infection rate was low and did not largely differ across the subgroups with different background (Table 2). During the Omicron epidemics, the infection rate has increased faster in younger age group, in doctors and nurses, and in participants who engaged in high risk of occupational exposure to SARS-CoV-2 than their counterparts.

**Table 2.** Chronological change of COVID-19 cumulative incidence according to background factors

	Seroepidemiological surveys						
Total cases, *n*/*N* (%)	1st (July 2020)	2nd (October/December 2020)	3rd (June 2021)	4th (December 2021)	5th (March 2022)	6th (June 2022)	7th (December 2022)
Sex							
Male	0/358 (0.0)	7/778 (0.9)	16/814 (2.0)	19/227 (8.4)	31/400 (7.8)	139/778 (17.9)	189/462 (40.9)
Female	3/870 (0.3)	12/1786 (0.7)	39/1956 (2.0)	31/716 (4.3)	101/1117 (9.0)	309/1875 (16.5)	450/1177 (38.2)
Age, years							
<30	2/460 (0.4)	8/803 (1.0)	26/920 (2.8)	26/495 (5.3)	45/441 (10.2)	172/832 (20.7)	242/514 (47.1)
30–39	0/338 (0.0)	1/630 (0.2)	12/701 (1.7)	11/246 (4.5)	35/382 (9.2)	129/671 (19.2)	178/414 (43.0)
40–49	1/259 (0.4)	4/593 (0.7)	10/615 (1.6)	8/144 (5.6)	36/374 (9.6)	95/578 (16.4)	136/361 (37.7)
≥50	0/171 (0.0)	6/538 (1.1)	7/534 (1.3)	5/58 (8.6)	16/320 (5.0)	52/572 (9.1)	83/350 (23.7)
Job							
Doctors	0/237 (0.0)	2/438 (0.5)	14/489 (2.9)	10/130 (7.7)	13/226 (5.8)	91/443 (20.5)	117/263 (44.5)
Nurses	2/601 (0.3)	8/971 (0.8)	26/1096 (2.4)	25/636 (3.9)	72/589 (12.2)	203/1016 (20.0)	288/652 (44.2)
Allied healthcare professionals	0/169 (0.0)	3/368 (0.8)	7/364 (1.9)	9/160 (5.6)	16/231 (6.9)	49/360 (13.6)	76/218 (34.9)
Administrative staff	1/128 (0.8)	2/290 (0.7)	2/264 (0.8)	2/5 (40.0)	10/165 (6.1)	38/289 (13.1)	52/175 (29.7)
Others	0/93 (0.0)	4/497 (0.8)	6/557 (1.1)	4/12 (33.3)	21/306 (6.9)	67/545 (12.3)	106/331 (32.0)
Occupational risk of SARS-CoV-2 infection^a^							
Low	3/408 (0.7)	13/1212 (1.1)	25/1468 (1.7)	23/434 (5.3)	77/858 (9.0)	240/1584 (15.2)	325/907 (35.8)
Moderate	0/478 (0.0)	3/712 (0.4)	12/708 (1.7)	10/232 (4.3)	29/324 (9.0)	92/575 (16.0)	161/384 (41.9)
High	0/342 (0.0)	3/619 (0.5)	17/572 (3.0)	17/274 (6.2)	26/335 (7.8)	112/487 (23.0)	151/346 (43.6)

a Number of missing values for the occupational SARS-CoV-2 exposure at the 1st to 7th surveys were 21, 22, 3, 0, 7 and 2, respectively.

## Discussion

During the first year of the pandemic, the seroprevalence of SARS-CoV-2 antibody among the NCGM staff was very low [7] (even lower than that among the general population), which could be ascribed to comprehensive measures against nosocomial infection including the use of personal protective equipment and strict adherence to infection preventive practices. In the present extended study, we found a marked increase of SARS-CoV-2 infection after the emergence of the Omicron variants. It is estimated that nearly 40% staff have contracted COVID-19 by December 2022.

Serosurveys in the general population of Japan also showed data suggesting a rapid expansion of the infection during the early period of the Omicron epidemic [12]; the proportion of those infected (defined as either seropositive on the Roche assay or having a history of COVID-19) among Tokyo residents has increased from 3.1% in December 2021 to 6.4% in February 2022. The corresponding figures in the present population (re-calculated using the same definition) were 4.3% in December 2022 and 8.8% in March 2022. Although direct comparison of these data is limited due to the difference in age, sex and the timing of the survey, there seems no measurable difference in the cumulative infection of SARS-CoV-2 between NCGM staff and Tokyo residents after the emergence of variants.

While the risk of nosocomial infection has been well managed in NCGM, the chance of extra-hospital contact with patients with COVID-19 might have greatly increased after the emergence of the Omicron variant. According to the in-house registry, household infection has become a major identifiable route of transmission after January 2022, in parallel with the surge of children with COVID-19. Previously, we showed that the staff living with younger school-age children had a three-times higher risk of absence due to COVID-19 during the early period of the Omicron-predominant epidemic, compared with those living without such children [13].

Of participants who tested seropositive or reported a history of COVID-19 at the survey in June 2022, 44% were not aware of their infection, a figure comparable to that for US healthcare employees during the Omicron epidemic [8]. The proportion of undiagnosed infections tended to decrease over time in the present study population: 60% or above in 2020 and 2021 surveys but 30% at the latest survey in December 2022. This could be due, at least in part, to the improved availability of and access to diagnostic tests for the staff including self-check programme using an antigen test kit. We also speculate that people may be more likely to ascribe symptoms to COVID-19 during a larger epidemic (due to the high pre-test probability), thus taking a diagnostic test.

Our study has strengths including repeated antibody measurements since the early period of the pandemic through the post-vaccine Omicron-variant era in a well-defined population of a relatively large size. We should also acknowledge the limitations of the study. While we measured SARS-CoV-2 antibody using assays with high sensitivity and specificity, such performance data were derived from validation studies where blood specimens used were from a mostly hospitalised population known to be very recently infected with SARS-CoV-2 [4]. It remains unclear whether the cutoffs determined and validated by those studies are appropriate in the detection of asymptomatic or milder forms of this infection. Another concern is the waning of antibody and thus lowering of sensitivity over time, depending on assay used and vaccine status. The Roche assay can detect antibodies over an extended period after infection, whereas the ability of the Abbott assay in detecting past infection decreases with time [14, 15]. In the present study, discordance in the seropositivity between the two assays has enlarged over time (Supplementary Table S2); at the last survey, the discordant pattern of Abbott (−) and Roche (+) (21.4%) was much higher than the opposite (Abbott (+) and Roche (−) = 0.7%), probably reflecting the faster decline in sensitivity of Abbott assay. Due to the lack of sex- and age-specific data from the serosurvey among Tokyo residents, these factors were not considered when comparing the cumulative infection between the present and general populations. The present study was done among staff of a medical research centre in Tokyo; thus, the results may not be applicable to the general population.

## Conclusion

In summary, the present extended study showed a rapid expansion of COVID-19 after the emergence of the Omicron variant among the staff of a medical research centre in Tokyo. As of December 2022, nearly 40% of the NCGM staff might have been infected. Given that past infection is a strong predictor for a lower risk of SARS-CoV-2 infection and severe COVID-19 [16], repeated serosurveys among healthcare workers could contribute not only to the monitoring the spread of COVID-19 including undiagnosed cases but also to the assessment of individual- and population-immunity towards the so-called ‘with corona’ era.

## Data Availability

The datasets generated during and/or analysed during the current study are available from the corresponding author on reasonable request.

## Supplementary material

For supplementary material accompanying this paper visit https://doi.org/10.1017/S0950268823000353. click here to view supplementary material
