# Supplementary material for: Cumulative and undiagnosed SARS-CoV-2 infection among the staff of a medical research centre in Tokyo after the emergence of variants
Source: Epidemiol Infect. 2023 Mar 8;151:e48. doi: 10.1017/S0950268823000353 (PMC10063865; doi:10.1017/S0950268823000353)
Supplement: Supplementary file 1 [file hygsup.zip › S0950268823000353sup001.docx]

**Key Findings**

- Serostudy was done to estimate the spread of COVID-19 among healthcare workers in Tokyo.

- Cumulative infection rate was estimated to be 5.3% after the Delta predominant epidemic.

- After the emergence of Omicron variant, it markedly increased to 39.0%, as of December 2022.

- Of those who might have infected in the past, 30% were not aware of their infection.
