# Supplementary material for: Cumulative and undiagnosed SARS-CoV-2 infection among the staff of a medical research centre in Tokyo after the emergence of variants
Source: Epidemiol Infect. 2023 Mar 8;151:e48. doi: 10.1017/S0950268823000353 (PMC10063865; doi:10.1017/S0950268823000353)
Supplement: Supplementary file 1 [file hygsup.zip › S0950268823000353sup002.docx]

**Supplementary Materials**

Table of Contents

[Table S1. The chronological change of COVID-19 indicators among the staff of the Toyama Hospital 1](#_Toc956376164)

[Table S2. The chronological change of the seropositivity using different definitions 2](#_Toc1698223871)

# **Table S1. The chronological change of COVID-19 indicators among the staff of the Toyama Hospital**

|  | Seroepidemiological surveys | | | | | | |
| --- | --- | --- | --- | --- | --- | --- | --- |
|  | 1st  (Jul. 2020) | 2nd  (Oct./Dec. 2020) | 3rd  (Jun. 2021) | 4th  (Dec. 2021) | 5th  (Mar. 2022) | 6th  (Jun. 2022) | 7th  (Dec. 2022) |
| Participants, N | 1,228 | 2,055 | 2,219 | 943 | 1,517 | 2,104 | 1,639 |
| Diagnosed cases^a^, n(%) | 1 (0.1) | 3 (0.1) | 15 (0.7) | 20 (2.1) | 73 (4.7) | 204 (9.7) | 449 (27.4) |
| Seropositive cases^b^, n(%) | 2 (0.2) | 16 (0.8) | 46 (2.1) | 48 (5.1) | 121 (8.0) | 353 (16.8) | 604 (36.9) |
| Total cases, n(%) | 3 (0.2) | 17 (0.8) | 47 (2.1) | 50 (5.3) | 132 (8.7) | 365 (17.3) | 639 (39.0) |
| Awareness^c^, % | 33.3 | 17.6 | 31.9 | 40.0 | 55.3 | 55.9 | 70.3 |
| Unawareness^d^, % | 66.6 | 82.4 | 68.1 | 60.0 | 44.7 | 44.1 | 29.7 |
| Ratio of total to diagnosed cases | 3.0 | 5.7 | 3.1 | 2.5 | 1.8 | 1.8 | 1.4 |
| Vaccination status, % |  |  |  |  |  |  |  |
| Unvaccinated | 100 | 100 | 8.7 | 2.8 | 1.6 | 2.1 | 1.2 |
| 1 or 2-dose | 0 | 0 | 91.3 | 63.2 | 2.9 | 4.4 | 2.8 |
| 3-dose | 0 | 0 | 0 | 34.0 | 95.4 | 92.1 | 29.4 |
| ≥4-dose | 0 | 0 | 0 | 0 | 0.1 | 1.3 | 66.5 |

^a^ Confirmed with PCR or antigen test and/or diagnosed by a physician without test

^b^ Positive on Abbott and/or Roche SARS-CoV-2 anti-nucleocapsid protein antibody assays

^c^ The proportion of diagnosed cases among total cases

^d^ The proportion of undiagnosed cases (seropositive only) among total cases

# **Table S2. The chronological change of the seropositivity using different definitions**

|  | Seroepidemiological surveys | | | | | | |
| --- | --- | --- | --- | --- | --- | --- | --- |
|  | 1st  (Jul. 2020) | 2nd  (Oct./Dec. 2020) | 3rd  (Jun. 2021) | 4th  (Dec. 2021) | 5th  (Mar. 2022) | 6th  (Jun. 2022) | 7th  (Dec. 2022) |
| Participants, N | 1,228 | 2,564 | 2,770 | 943 | 1,517 | 2,653 | 1,639 |
| Abbott (+) | 1 (0.1) | 10 (0.4) | 21 (0.8) | 17 (1.8) | 80 (5.3) | 205 (7.7) | 254 (15.5) |
| Roche (+) | 1 (0.1) | 13 (0.5) | 43 (1.6) | 39 (4.1) | 116 (7.6) | 411 (15.5) | 593 (36.2) |
| Abbott (+) or Roche (+) | 2 (0.2) | 18 (0.7) | 54 (1.9) | 48 (5.1) | 121 (8.0) | 436 (16.4) | 604 (36.9) |
| Abbott (+) and Roche (+) | 0 (0.0) | 5 (0.2) | 10 (0.4) | 8 (0.8) | 75 (4.9) | 180 (6.8) | 243 (14.8) |
| Abbott (+) and Roche (-) | 1 (0.1) | 5 (0.2) | 11 (0.4) | 9 (1.0) | 5 (0.3) | 25 (0.9) | 11 (0.7) |
| Abbott (-) and Roche (+) | 1 (0.1) | 8 (0.3) | 33 (1.2) | 31 (3.3) | 41 (2.7) | 231 (8.7) | 350 (21.4) |
